# Genome wide expression profiling of p53 regulated miRNAs in neuroblastoma

**DOI:** 10.1038/srep09027

**Published:** 2015-03-12

**Authors:** Ali Rihani, Alan Van Goethem, Maté Ongenaert, Sara De Brouwer, Pieter-Jan Volders, Saurabh Agarwal, Katleen De Preter, Pieter Mestdagh, Jason Shohet, Frank Speleman, Jo Vandesompele, Tom Van Maerken

**Affiliations:** 1 Center for Medical Genetics, Ghent University, De Pintelaan 185, B-9000 Ghent, Belgium; 2 Texas Children's Cancer Center, Baylor College of Medicine, Houston, Texas, USA

## Abstract

Restoration of the antitumor activity of p53 could offer a promising approach for the treatment of neuroblastoma. MicroRNAs (miRNAs) are important mediators of p53 activity, but their role in the p53 response has not yet been comprehensively addressed in neuroblastoma. Therefore, we set out to characterize alterations in miRNA expression that are induced by p53 activation in neuroblastoma cells. Genome-wide miRNA expression analysis showed that miR-34a-5p, miR-182-5p, miR-203a, miR-222-3p, and miR-432-5p are upregulated following nutlin-3 treatment in a p53 dependent manner. The function of miR-182-5p, miR-203a, miR-222-3p, and miR-432-5p was analyzed by ectopic overexpression of miRNA mimics. We observed that these p53-regulated miRNAs inhibit the proliferation of neuroblastoma cells to varying degrees, with the most profound growth inhibition recorded for miR-182-5p. Overexpression of miR-182-5p promoted apoptosis in some neuroblastoma cell lines and induced neuronal differentiation of NGP cells. Using Chromatin Immunoprecipitation-qPCR (ChIP-qPCR), we did not observe direct binding of p53 to *MIR182*, *MIR203*, *MIR222*, and *MIR432* in neuroblastoma cells. Taken together, our findings yield new insights in the network of p53-regulated miRNAs in neuroblastoma.

Under normal physiological conditions, MDM2 inhibits p53 by binding to its transcriptional activation domain^1^ and by promoting its degradation via an E3-ubiquitin ligase activity^2^ maintaining low steady-state levels of p53 expression. In response to various intrinsic or extrinsic stress signals, p53 is relieved from MDM2 inhibition leading to activation of the p53-controlled program of cell cycle arrest, cellular senescence or apoptosis. The p53 transcription factor controls a transcriptional network of p53-responsive genes and non-coding RNAs that collectively drive a given cellular response^1^,^3^. New insights into the mechanisms by which p53 regulates cellular growth/apoptosis/senescence can be gained by identifying up or downregulated microRNAs (miRNAs) upon p53 activation.

MiRNAs are small non-coding RNAs of 18–23 nucleotides in length that regulate gene expression at the post-transcriptional level mainly by binding in a sequence specific manner to the 3′-untranslated regions (3′UTRs) of messenger RNAs (mRNAs) and negatively regulating their expression^2^,^4^. MiRNAs have been shown to be an integral component of the p53 pathway regulating multiple p53-controlled biological processes^5^. Altered expression of tumor suppressive or oncogenic miRNAs can disrupt the p53-miRNA axis enhancing tumor growth or decreasing tumor proliferation. Although several miRNAs such as the miR-34 family^6^, miR-145^7^, miR-107^8^, miR-192, and miR-215^9^ have been shown to be essential components of the p53 tumor suppressor network, the spectrum of p53 regulated miRNAs in neuroblastoma remains to be established in detail.

Neuroblastoma is the most common extra-cranial solid childhood cancer. Although less than 2% of neuroblastoma tumors diagnosed harbor a *TP53* (*p53*) mutation, p53 fails to act as an effective tumor suppressor^10^. In consideration of the fact that the paradigm of cancer treatment shifts from broadly acting genotoxic agents to biologically targeted therapies, the prospect of targeting MDM2 to reactivate p53 holds promise for the molecular therapy of neuroblastoma. A small molecule antagonist of MDM2, nutlin-3, can restore p53 function by selectively disrupting the interaction between MDM2 and p53. Consequently p53 accumulates and induces the expression of its target genes. We have previously shown that nutlin-3 has profound effects on neuroblastoma cells and xenografts leading to premature senescence, apoptosis, and neuronal differentiation^2^,^11^.

In this study we performed a global megaplex profiling of 750 miRNAs in neuroblastoma cells after p53 activation and subsequently identified differentially expressed miRNAs. A neuroblastoma cell line lentivirally transduced with a short hairpin RNA against human p53 or murine p53 (negative control) was used to identify the p53-dependent nature of the miRNA expression alterations. We report here that four miRNAs (miR-222-3p, miR-432-5p, miR-182-5p, and miR-203a) are upregulated after p53 activation in neuroblastoma cells. Using the xCELLigence Real Time Cell Analyzer (RTCA) we demonstrate that overexpression of these miRNAs inhibits neuroblastoma cell growth to varying degrees as compared to neuroblastoma cells transfected with scrambled pre-miR. MiR-182-5p displayed the strongest growth-suppressive activity, had pro-apoptotic activity in some neuroblastoma cell lines and induced neuronal differentiation of NGP cells.

## Results

### Nutlin-3 induces p53 regulated miRNAs and p53 responsive genes

**Figure 1:**
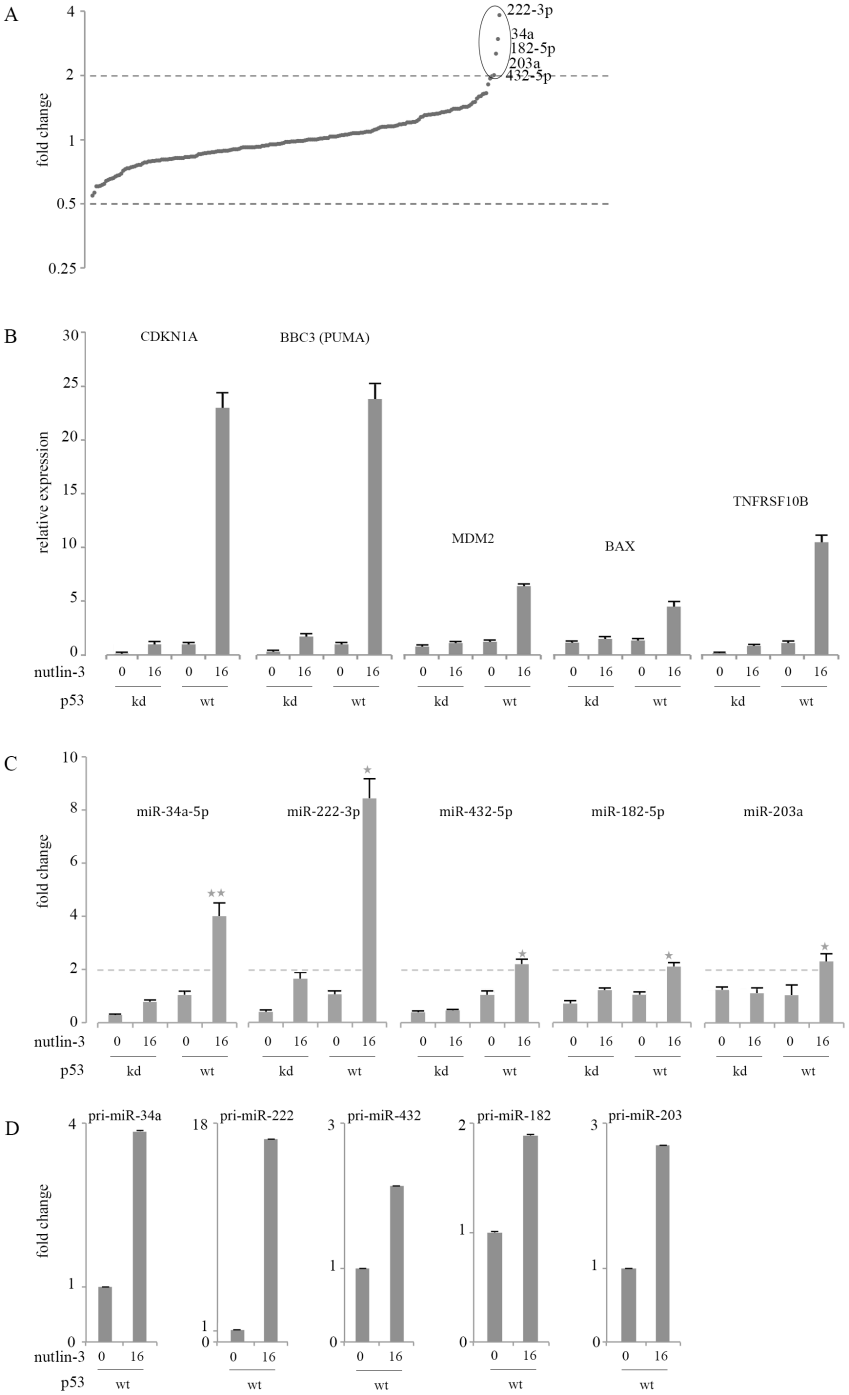
Nutlin-3 induces p53-regulated miRNAs and genes. (A), NGP cells were treated with 0 or 16 μM nutlin-3 for 24 hours. Expression fold changes of 750 miRNAs was determined using megaplex stem-loop RT primers and miRNA RT-qPCR. The scatter plot represents the average fold change expression values of expressed miRNAs in NGP cells after nutlin-3 treatment. miRNAs that demonstrated a linear fold change larger than 2 (>2-fold upregulation) or lower than 0.5 (<2-fold downregulation) were considered for further analysis. miR-222-3p, miR-34a-5p, miR-182-5p, miR-432, and miR-203a were upregulated and no miRNA was found to be downregulated. (B), p53 target genes are selectively induced in the control cells upon nutlin-3 treatment. NGP-LV-hp53 (kd) and NGP-LV-mp53 (wt) cells were treated with 0 or 16 μM nutlin-3 for 24 hours with the ethanol (solvent) concentration kept constant. Results are RT-qPCR normalized relative mRNA expression values (linear scale, mean of 2 reactions ± SEM) for direct p53 target genes: *CDKN1A*(*p21*), *MDM2*, *BBC3* (*PUMA*), *TNFRSF10B*, and *TP53I3*. (C), NGP-LV-hp53 (kd) and NGP-LV-mp53 (wt) cells were treated with 0 or 16 μM nutlin-3 for 24 hours. Relative expression of miR-34a-5p, miR-222-3p, miR-432-5p, miR-182-5p, and miR-203a was determined by miRNA TaqMan assays in a multiplex primer pool. Data represents the average relative miRNA expression of three independent biological replicates (linear scale, mean ± SEM, one-tailed Student *t*-test: *P < 0.05, **P < 0.001). miR-500. miR-503, and miR-484 were the most stable miRNAs from the megaplex profiling and were used as internal controls in the multiplex pool for normalization purposes. Dotted line represents the 2-fold threshold. (D), NGP cells treated with 0 or 16 μM nutlin-3 for 24 hours. Expression of pri-miR-34a, pri-miR-222, pri-miR-432, pri-miR-182, and pri-miR-203 was determined using primary miRNA TaqMan assays.

The p53 protein is known to activate the transcription of a subset of miRNA-coding genes^1^,^3^,^5^; however, little is known about the p53-regulated miRNAs in neuroblastoma. To identify p53-regulated miRNAs in neuroblastoma cells we treated the human p53 wild-type neuroblastoma cell line NGP with 0 or 16 μM nutlin-3 for 24 hours with two independent biological replicates. Subsequently, we performed a whole genome miRNA expression profiling using TaqMan based miRNA expression platform, and calculated the average miRNAs expression fold changes between treated and untreated cells with +/− 2× as a linear fold change cut-off value. Our results confirm that miR-34a-5p is upregulated after nutlin-3 treatment (figure 1A) in line with what is reported about mir-34a-5p as a direct target of p53^2^,^4^,^5^. Besides miR-34a-5p upregulation, miR-222-3p, miR-182-5p, miR-203a and miR-432-5p are upregulated as well. No miRNAs were found to be downregulated by crossing the 0.5 fold change. To assess whether the upregulation of the aforementioned miRNAs is specifically mediated by p53, we used NGP cells lentivirally transfected with a short hairpin RNA against human TP53 (NGP-LV-hp53) or murine TP53 (NGP-LV-mp53)^5^,^11^. The cells were treated in the same way by 0 or 16 μM nutlin-3 for 24 hours. The model system was first validated by analyzing the expression of 6 direct p53 target genes using RT-qPCR (figure 1B). The basal expression of these genes was higher and increased further upon nutlin-3 treatment in NGP-LV-mp53 cells (control) as compared to the NGP-LV-hp53 cells (*p53*-knock down).

Next we confirmed the upregulation of these selected miRNAs by multiplex RT-qPCR (figure 1C). In general, the basal expression of the miRNAs was higher and increased upon nutlin-3 treatment in the control cells as compared to the NGP-LV-hp53 cells (p53-KD). MiR-222-3p was found to be upregulated by more than 8-fold in response to nutlin-3 treatment, whereas miR-34a-5p showed 4-fold upregulation. MiR-432-5p, miR-203a, and miR-182-5p showed 2-fold upregulation (figure 1C).

p53 has been shown to enhance the processing of precursor miRNAs^5^. We measured the expression of the precursor miRNAs using TaqMan based assays. Our results show that the expression of the precursor miRNAs has a similar pattern to the expression of the mature miRNAs, suggesting that the regulation of these miRNAs is at the transcriptional level (figure 1D).

Our results show that miR-222-3p, miR-182-5p, miR-203a and miR-432-5p are upregulated in neuroblastoma cell lines after p53 activation by nutlin-3.

### miR-182-5p inhibits the proliferation of neuroblastoma cells

**Figure 2:**
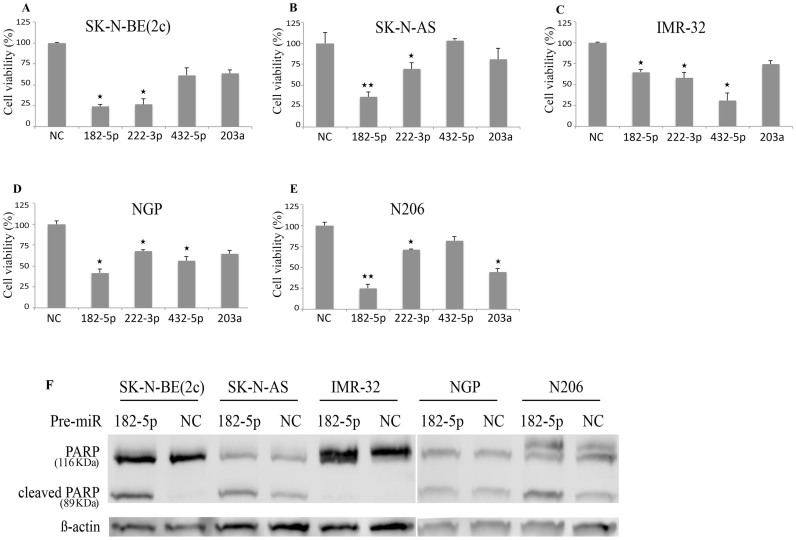
Overexpression of miRNAs reduces proliferation of neuroblastoma cells. Neuroblastoma cell lines were transfected with the indicated pre-miRs, and their growth was assessed using the xCELLigence system. The data shown represents the percentage of cell viability of the neuroblastoma cell lines 96 hours post transfection with the indicated pre-miRs. Two-tailed paired t-test was used to compare the viability of the neuroblastoma cell lines transfected with the pre-miRs to the negative control *q (corrected *p*-value) < 0.05, ** *q* < 0.001). IMR-32 (A), SK-N-AS (B), NGP (C), SK-N-BE(2c) (D), N206 (E). Protein expression of full length PARP (upper band) and cleaved PARP (lower band) in SK-N-BE(2c) cells 48 hours post transfection with pre-miR-222-3p or pre-miR-NC (non-targeting control). β-actin was used as a loading control (F).

To gain insight into the effect of ectopic overexpression of miR-222-3p, miR-203a, miR-182-5p, and miR-432-5p on the proliferation of neuroblastoma cells, we transfected 5 cell lines with pre-miRs (miRNA mimics) and evaluated the cell growth in real time using the xCELLigence system. Two *p53*-wild type (NGP and IMR-32) and three *p53*-mutant (SK-N-BE(2c), N206, and SKNAS) neuroblastoma cell lines were used. The cells were seeded in duplicates and transfected with 100 nM of scrambled pre-miR serving as a negative miRNA control (pre-miR-NC), or pre-miR precursor molecule for miR-222-3p, miR-203a, miR-182-5p, and miR-432-5p. MiR-182-5p shows the most prominent inhibition of the proliferation of neuroblastoma cells (figure 2A–2E). The real time viability data of the tranfection with the four miRNA mimics is shown in figure S1. Furthermore, miR-182-5p showed pro-apoptotic activity, as demonstrated by its ability to induce PARP cleavage in SK-N-BE(2c) cells and to a lesser extent also in SK-N-AS and N206 cells (figure 2F).

In short, our results show that overexpression of the p53-regulated miRNAs inhibit the proliferation of neuroblastoma cells to varying degrees. In addition, overexpression of miR-182-5p can promote apoptosis, as demonstrated by PARP cleavage.

### miR-182-5p upregulation induces differentiation of NGP cells

**Figure 3:**
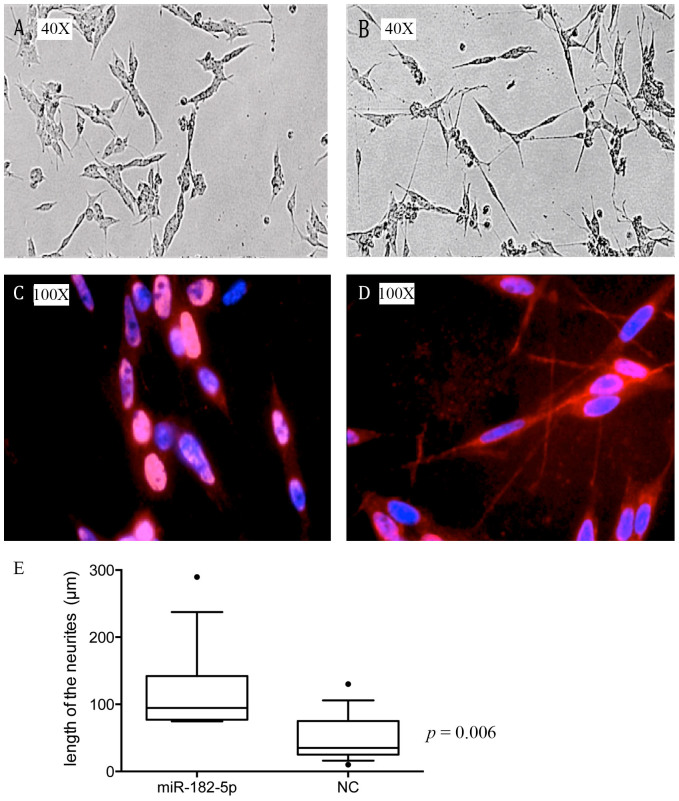
miR-182-5p induces neurite outgrowth in NGP cells. NGP cells were transfected with pre-miR NC (A,C), or pre-miR-182-5p (B,D). Neurofilament staining was done 5 days post transfection. The upper panel (A,B) shows the morphology of cells before staining. Nuclei were stained with blue fluorescent DAPI, and red fluorescence for neurofilaments with Cy3 anti-neurofilament antibody (C, D). Box plots showing the length of the neurites in μm, the horizontal line represents the median, the box represents the interquartile range, the whiskers represent 10–90 percentile, and the dots represent the outliers (E).

We previously reported that nutlin-3 treatment may induce differentiation of certain neuroblastoma cells^6^,^11^ and thus hypothesized that p53 upregulated miRNAs can act as positive mediators of this differentiation response. To elucidate the effect of ectopic overexpression of the four miRNAs on cellular differentiation, we performed neurofilament staining 5 days post pre-miR transfection in NGP cells. In short, NGP cells were transfected with mature microRNA mimics or a negative control. Overexpression of miR-182-5p clearly induced neurite outgrowth 5 days post transfection (figure 3). The other miRNAs did not induce neuroblastoma differentiation.

### p53 does not bind to *MIR182*, *MIR203*, *MIR222*, and *MIR432* in neuroblastoma cells

**Figure 4:**
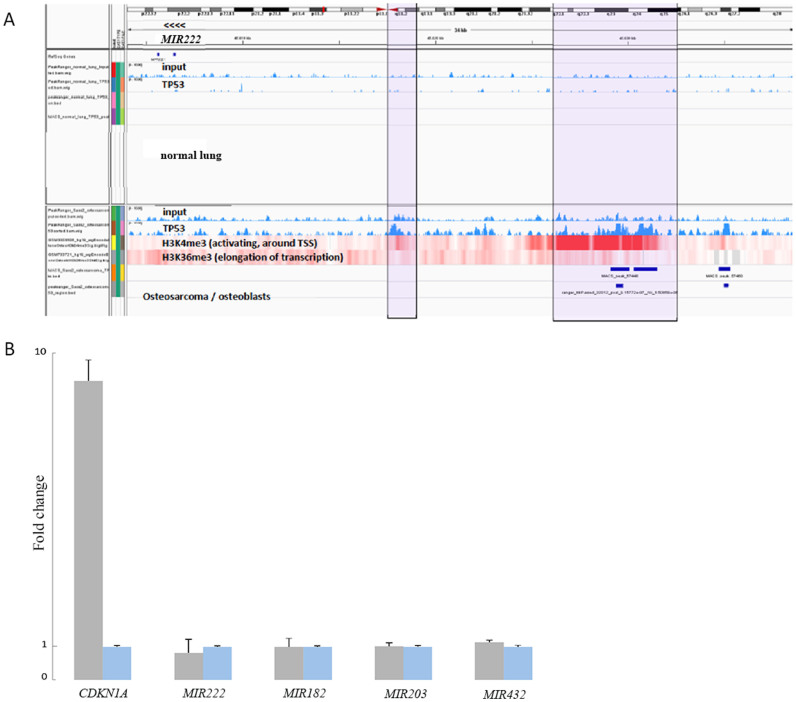
p53 ChIP-Seq and ChIP-qPCR. IGV visualization of MACS ChIP peak results using p53 ChIP-Seq data from normal lung fibroblast cells (IMR-90; upper part) and osteosarcoma cells (Saos2; lower part). Chromosome X is shown at the top of the figure with a red marker representing the location of miR-222-3p at Xp11.3. From top to bottom: peaks identified by MACS represent the pileup signal of the input (pre-ChIP) sample after mapping the reads to the sequence in the top of the figure. The input sample track shows non-specific background. The next track represents the peaks of the mapped reads using p53 antibody (p53 ChIP-Seq) after subtracting the non-specific background signal of the input sample. The lower part represents the signal from H3K4me3 data that marks the start of active transcription; and the signal from H3K36me3 data that marks the transcription elongation (A). ChIP-qPCR performed on p53-ChIP material from MYCN3, a *p53*-wild type neuroblastoma cell line. *CDKN1A* is a positive control p53 target gene (B).

In an attempt to characterize the mechanism of the p53 regulation of miRNAs, we used publicly available TP53 chromatin immunoprecipitation - sequencing (ChIP-Seq) data from IMR-90 normal lung fibroblast cells and Saos-2 osteosarcoma cells (GEO GSM783262 and GSM501692, respectively). We also used preprocessed data of chromatin marks for osteoblasts. The data was used to identify potential binding sites of p53 in the transcription start site of the miRNAs upregulated upon p53 activation. Using Model-based Analysis of ChIP-Seq (MACS)^8^,^12^ we identified enriched regions of p53 binding sites in the transcription start sites of *MIR222* (figure 4A). These data suggest that *MIR222* could be a direct target of p53, at least in these sample types. However, qPCR on p53-ChIP material from a neuroblastoma cell line with wild-type p53 (MYCN3) treated with nutlin-3 could not confirm direct binding of p53 to *MIR182*, *MIR203*, *MIR222*, and *MIR432*, whereas p53 binding to a positive control p53 target gene (*CDKN1A*) was clearly shown (figure 4B). In short, p53 appears to induce the expression of these miRNAs indirectly and not through direct binding to their promoter region.

## Discussion

MiRNAs are well known to be differentially expressed in tumors as compared to normal tissues^9^,^13^. Characterizing this differential expression may help us to understand better how tumors evade the apoptotic machinery, and proceed through the cell cycle and consequently cell growth and survival. As a guardian of the genome, p53 represents a central node that controls the decisions of either executing the apoptosis program or inducing survival and proliferation^10^,^14^. In consideration of the fact that less than 2% of neuroblastoma tumors harbor a mutation in *TP53* at diagnosis^10^, it becomes reasonable to probe the p53 pathway and identify the factors that assist neuroblastoma tumors to sustain their growth potential and evade p53 pathway suppressing effects. Expression changes in 15% of p53 pathway genes after p53 activation in response to stress signals is attributable to miRNAs^5^. In this study, we identify p53-regulated miRNAs via global expression profiling of 750 miRNAs in the *MDM2*-amplified neuroblastoma NGP cells after nutlin-3 treatment. Using NGP cells lentivirally transfected with a short hairpin RNA against human *TP53* (NGP-LV-hp53) or murine *TP53* (NGP-LV-mp53), we were able to confirm and validate the upregulation four miRNAs (miR-222-3p, miR-432-5p, miR-203a, and miR-182-5p) by p53 in neuroblastoma cells. We applied a strict selection criterion and excluded all miRNAs that had less than 2-fold differential expression. In addition to these four upregulated miRNAs, miR-34a-5p was also found to be upregulated. *MIR34A* is located at 1p36, a frequently deleted region in neuroblastoma tumors and has been characterized as a tumor suppressor gene in neuroblastoma tumors^15^. In addition, *MIR34A* has already been reported to be a direct target of p53^6^. As miR-34a-5p has already been studied extensively in literature, we did not focus in our functional analysis on this miRNA. Nevertheless, observed upregulation of miR-34a-3p and miR-34a-5p in NGP cells after p53 activation is a nice positive control.

We investigated the functional effects of miR-222-3p, miR-432-5p, miR-203a, and miR-182-5p in *p53*-wild type and *p53*-mutant neuroblastoma cell lines. Our results showed that ectopic overexpression of these miRNAs using miRNA mimics reduced the growth of the neuroblastoma cell lines, with the most pronounced effects being induced by miR-182-5p. Pro-apoptotic activity of miR-182-5p was documented by demonstrating its ability to induce PARP cleavage in SK-N-BE(2c) cells and to a lesser extent also in SK-N-AS and N206 cells.

In addition, miR-182-5p induced neuronal differentiation of NGP cells. MiR-182-5p has already been shown to be upregulated after p53 activation in several cancer entities such as colon cancer^6^, lung cancer^16^, and uveal melanoma^17^. In addition, p53 has been reported to enhance the post-transcriptional maturation of miR-182-5p^18^. These data support our findings on the regulation of miR-182-5p by p53.

Of note, miR-182-5p has been reported to inhibit the growth of melanoma cell lines^19^ and the proliferation of gastric adenocarcinoma cell lines^20^. However, miR-182-5p was also reported to be a potent anti-apoptotic miRNA in colon cancer^21^ and in hepatocellular carcinoma^22^, suggesting a tissue-specific effect.

It is worth mentioning that the other p53-regulated miRNAs have also already been implicated in cancer. For example, miR-222-3p functions as an oncogene in some tumors and as a tumor suppressor in others^10^,^23^,^24^, which also suggests that the function of miR-222-3p is tumor and cellular context dependent.

*MIR203* and *MIR432* are located at 14q32.33 and 14q32.31 respectively, a frequently deleted region in neuroblastoma tumors^23^. Upregulation of miR-203a was shown to be dependent on p53 activation in keratinocytes^25^, and p53 was also reported to enhance the post-transcriptional maturation of miR-203a^18^, and it has been described intensively as a tumor suppressor in several cancer entities^26^,^27^,^28^.

Little information exists about miR-432-5p in literature. This miRNA has been reported to play a tumor suppressive role in pituitary adenocarcinoma^29^ and to be highly upregulated during senescence of fibroblasts^30^ and during the earliest stages of fetal development^31^. We analysed the expression of miR-432-5p in neuroblasts and neuroblastoma samples. We found that miR-432-5p expression is associated with progression free survival of neuroblastoma patients and that this miRNA is expressed at higher levels in neuroblasts as compared to neuroblastoma tumors (figure S2).

## Conclusion

We have identified four p53-regulated miRNAs, which inhibit the growth of neuroblastoma cells. The most profound inhibition of proliferation was observed for miR-182-5p, which was shown to have pro-apoptotic activity in some neuroblastoma cell lines and to induce neuronal differentiation of NGP cells. Further research is likely to add more miRNAs to the growing list of p53-regulated miRNAs, and further functional assays are required to decipher the molecular mechanisms underlying the p53-mediated cellular responses.

## Methods

### Cell lines and nutlin-3 treatment

Human neuroblastoma cell lines used in this study are: NGP, NGP-lv-hp53, NGP-lv-mp53, SK-N-AS, SK-N-Be(2c), IMR-32, IMR-32-lv-hp53, IMR-32-lv-mp53. NGP/IMR-lv-hp53 and NGP/IMR-lv-mp53 are cell lines lentivirally transfected with a short hairpin RNA against human p53 or murine p53 (control), respectively^11^. Cells are grown as monolayer cultures at 37°C and 5% CO_2_ in a humid atmosphere. The culture medium is complete RPMI 1640 (GIBCO, Life Technologies) containing 10% Foetal Calf Serum (FCS), and the following antibiotics unless stated otherwise: Penicillin (1%), Kanamycin (1%), Streptomycin (1%) and 2 mmol/l glutamine. Nutlin-3 (Cayman Chemical) was dissolved in ethanol and stored as a 10 mM stock solution at −20°C. Keeping the final concentration of ethanol constant, cells were treated with nutlin-3 ranging from 0 to 16 μM for the time periods indicated. Calculation of the volumes needed for the nutlin-3 treatment was done using the online calculator: www.calculators.alirihani.com.

### RNA extraction

Cells were harvested by scraping and total RNA was extracted using miRNeasy Micro Kit (Qiagen, Hilden) with RNase-free DNase I treatment performed on RNA extraction spin column according to the manufacturer's instructions. RNA concentration was measured using the Nanodrop UV spectrophotometry platform (Nanodrop Technologies, USA). RNA integrity for all samples was assessed using Experion (software version 3.2, Bio-Rad).

### Megaplex/multiplex miRNA reverse transcription, pre-amplification and expression quantification

Reverse transcription of 750 miRNAs was done using reverse transcription kit (Applied Biosystems) and stem-loop Megaplex primer pools (Applied Biosystems). In short, 80 μl reaction volume was prepared by adding 8 μl of total RNA (50 ng/μl), RT primer mix (10×), RT buffer (10×), Multiscribe Reverse Transcriptase (10 U/μl), dNTPs with dTTP (0.5 mM each), MgCl_2_ (3 mM) and AB RNase inhibitor (0.25 U/ml). A pulsed RT reaction was then performed with 40 cycles of 16°C for 2 min, 42°C for 1 min, and 50°C for 1 s. These 40 cycles were followed by increasing the temperature to 85°C for 5 minutes to inactivate the reverse transcriptase. Afterwards, the RT product was pre-amplified in a 25 μl PCR reaction using Applied Biosystems' TaqMan PreAmp Master Mix (2×) and PreAmp primer mix (5×). The primer pool consisted of a universal reverse primer (50 nM) (Applied Biosystems) and 750 forward primers (50 nM) specific for each miRNA. The following pre-amplification cycling conditions were used: 95°C for 10 min, 55°C for 2 min, and 75°C for 2 min followed by 14 cycles of 95°C for 15 s and 60°C for 4 min. Real-time qPCR was performed using the 7900HT RT-qPCR system (Applied Biosystems) in 8 μl reaction consisting of 4 μl of TaqMan Master Mix (Applied Biosystems), 1 μl of cDNA, and 3 μl of miRNA TaqMan probe and primers (Applied Biosystems). The following cycling conditions were applied: 95°C for 10 min followed by 40 cycles of 95°C for 15 s and 60°C for 1 min. Raw Cq values were determined using SDS 2.1 software with automated baseline settings and 0.2 treshold value (see Ref. 32 for more details). Only miRNAs with Cq values of 32 or below were considered for further analysis. The relative expression of miRNAs was normalized using mean expression values of all expressed miRNAs in every sample according to Mestdagh et *al*.^33^.

To validate the megaplex miRNA profiling results; we performed multiplex by following the same protocol described above with slight modifications. Briefly RNA was reverse transcribed using multiplex RT primer pools containing miRNA-specific stem-loop primers for the miRNAs in question. No pre-amplification was done, and the total volume of RT-qPCR reaction was 5 μl. Normalization was done in qbase+ using the geometric mean expression value of the most stably expressed miRNAs identified after the megaplex profiling. Measuring the miRNAs precursors was done using stem-loop TaqMan assays. The cDNA was prepared using the high capacity RT kit (Applied Biosystems) adding 50 ng of total RNA, RT buffer (2×), RT enzyme mix (20×) and RNase-free water to a reaction volume of 20 μl. A pulsed RT reaction was then performed as described above. qPCR was then performed according to the manufacturer's protocol using 10 ng of the RT product, TaqMan universal master mix (2×) and TaqMan Pri-miRNA assays (20×) (Applied Biosystems) in 8 μl reaction volume. The following cycling conditions were applied: 95°C for 10 min followed by 40 cycles of 95°C for 15 s and 60°C for 1 min.

### Pre-miR transfection

Neuroblastoma cells were transfected with individual pre-miR molecules (Ambion) at a concentration of 100 nM using DharmaFECT 2 transfection reagent (Thermo Scientific). Pre-miR negative control 2 (Ambion) was used as a scrambled control. Cell were seeded in RPMI (Invitrogen) with 10% FCS and without antibiotics.

### Cell growth assessment

xCELLigence MP device from Roche Diagnostics was used to monitor cell proliferation in real-time. This system measures electrical impedance on the bottom of tissue culture electronic microtiter plates (E-Plate; Roche Diagnostics). The signal from the interdigitated electrodes at the bottom of every well is measured as the cell index. Prior to seeding, background impedance was determined using 40 μl of media (RPMI) containing 10% FCS and always subtracted as blank value. 1 × 10^4^ cells in 50 μl of RPMI containing 10% FCS were seeded in duplicates. The experiment was repeated three times. Cell proliferation was measured with a programmed signal detection every 15 min and the signal was normalized to the transfection time point where the cell index at every time point was divided by the cell index at the time of transfection. Data acquisition and analysis was performed with the RTCA software (version 1.2, Roche Diagnostics).

### RT-qPCR

Relative expression of protein coding genes was performed by RT-qPCR according to MIQE guidlines^34^. Briefly, cDNA synthesis was performed on the isolated RNA using iScript cDNA synthesis kit (Bio-Rad) according to the manufacturer's instructions.

RT-qPCR was done in duplicates in 5 μl reaction volume containing 2 μl (5 ng) of template cDNA, 2.5 μl of 2× Sso Advanced reaction mix (Bio-Rad), 0.25 μl of a 5 μM solution of each primer. LightCycler 480 (Roche) was used with the following cycling conditions: 10 s at 95°C followed by 45 cycles of denaturation (10 s at 95°C) and elongation (45 s at 60°C). Primers were designed using RTPrimerDB^35^. Biogazelle's qbase+ qPCR data-analysis software^36^ was used to quantify the relative expression of genes (http://www.qbaseplus.com). Most stably expressed reference genes selected from a panel of 10 commonly used reference genes were used for normalization. Primer sequences are available in the RTPrimerDB database (http://www.rtprimerdb.org): ACTB (RTPrimerDB ID #1), B2M (#2), GAPDH (#3), HMBS (#4), HPRT1 (#5), RPL13A (#6), SDHA (#7), UBC(#8), YWHAZ (#9). The sequence of the Alu repeats primers are CATGGTGAAACCCCGTCTCTA for the forward primer and GCCTCAGCCTCCCGAGTAG for the reverse primer.

### Western blot

Cells transfected with pre-miR-222 or pre-miR-NC for 48 hours were harvested and washed using ice cold PBS, centrifuged and the supernatant discarded. The pellet was solubilized in RIPA lysis buffer (Pierce) containing protease and phosphatase inhibitor mixture (Roche). Cell lysates were placed on ice for 30 minutes and centrifuged for 10 minutes at 12,000 rpm at 4°C. The protein concentration was measured using the Bio-Rad Protein Assay (Bio-Rad). Protein samples were mixed at 1:1 ratio with Laemmli denaturation buffer (Bio-Rad) and β-mercaptoethanol (Sigma Aldrich) at a final dilution of 1/40 and boiled for 10 minutes at 95°C. Approximately 25 μg of protein was loaded and fractionated using a 10% SDS-PAGE gel (Bio-Rad). The protein was transferred onto a nitrocellulose membrane (Bio-Rad) and immunoblotted with antibodies against cleaved PARP (Cell Signaling Technology) and β-actin (Sigma Aldrich) as a loading control. Secondary antibody was anti-mouse HRP-linked (Cell Signaling Technology). Visualization of the proteins was done using the ChemiDoc-It Imaging System (UVP).

### *In Vitro* Differentiation assay

Immunocytochemistry was performed to detect differentiation of NGP cells. Briefly, 10^5^ NGP cells in 1.9 ml of cell suspension with 10% FCS and without antibiotics were seeded in 6-well plates containing heat sterilized circular coverslips. 24 hours later, the cells were transfected as described above, and kept in an incubator at 37°C. After 4 days the 6-well plates were placed on ice for 30 min, and then culture medium was aspirated from each well and the cells were rinsed gently in phosphate buffered saline (PBS) (Invitrogen). The cells were then fixed by incubating them for 20 min in 1 ml PBS (pH = 7.4) containing-paraformaldehyde (3.7%) and subsequently incubated in 1 ml NH_4_Cl (50 mM) for 10 min on ice. Fixed cells were washed with PBS and permeabilized with ice cold acetone for 10 min. Thereafter, permeabilized cells were washed with PBS and blocked by incubating them on ice for 20 min in 1 ml PBS containing Fish Skin Gelatin (0.2%). The cells were then incubated with undiluted mouse monoclonal anti-neurofilament (NE-14) antibody (BioGenex) for 1 hour at room temperature. Following, the cells were washed with PBS and incubated with Cyanine 3 (Cy3)-conjugated secondary anti-mouse antibody for 1 hour at room temperature. Cells were then washed with PBS and transferred onto a slide. A droplet of 4′,6′-diamidino-2-phenylindole (DAPI) was added to stain the nuclei. The cells were finally visualized using Zeiss Axioplan 2 imaging analysis system (Carl Zeiss) equipped with an HBO-100 W Hg vapor lamp, 63.0 × 1.25 oil-immersion objective and appropriate filter sets for DAPI and Cy3 dyes. The images were captured by a digital camera (JAI corporation) and analyzed by Isis FISH Imaging System software V5.1 (MetaSystems GmbH).

### ChIP-Seq data analysis

Raw p53 ChIP-Seq data was downloaded from Gene Expression Omnibus (GEO)-GSM783262 (normal lung fibroblasts-IMR-90) and GSM501692 (osteosarcoma-Saos-2). Reads from the raw data were aligned using Burrows-Wheeler Alignment tool (BWA)^37^, and the peaks were called using MACS software^12^. ChIP tracks showing p53 binding sites were visualized using Integrative Genomics Viewer (IGV)^38^.

### ChIP qPCR

ChIP was performed on 1 × 10^7^ MYCN3 neuroblastoma cells either treated or untreated with nutlin3a for 8 h using ChIP-IT Express Chromatin Immunoprecipitation Kit (Active Motif) according to manufacturer's instructions. Samples were sonicated for 20 cycles of 30 sec intervals in a Bioruptor UCD-200 sonicator (Diagenode). CHIP was performed with Negative control Ab (control IgG) and anti-human p53 antibody and all the samples were validated by performing ChIP-qPCR using specific primers of p53 binding site at p21 promoter −2 kb site (EpiTect Chip ChIP-Grade Antibody Kit (p53), SABiosciences). Input was generated by purifying DNA from the sonicated lysates of each sample.

The identification of the miRNAs transcription start site (TSS) was based on a study by Ozsolak et al.^39^. The ChIP-qPCR primers listed in table S1 were designed using -Primer 3^40^ and were validated for specificity using Bowtie as an alignment tool^41^, and the SNPs were filtered out using the dbSNP database^42^. RT-qPCR was performed as described above using 4 ng of the eluted DNA material. Dissociation curves were analyzed for each primer pair as a means to ensure the quality of amplicon and to monitor primer dimers. The ChIP-qPCR data was analyzed according to the fold enrichment method where the data was normalized to multiple non-specific genomic regions (not bound by p53) and analyzed relative to an input sample.

### Statistical methods

For the survival analysis, patients were divided into four quartiles according to the expression status of miR-432 in their neuroblastoma tumor (low expression to high expression). Comparison of Kaplan-Meier survival curves between different groups was done using the log-rank test. *p*-values < 0.05 were considered statistically significant. All statistical analyses were conducted using R version 3.0.2 using “stats” statistical package.

Comparison of the length of the neurites between NGP cells transfected with pre-miR-182-5p and NGP cells transfected with negative control was done using the unpaired t-test in SPSS version 20 software.

Two-tailed paired t-test was used to compare the viability of the neuroblastoma cell lines transfected with the pre-miRs to the negative control. The test was done in SPSS version 20 software on the data of two biological replicates (two experiments with two replicates per condition). This analysis was followed by Benjamini-Hochberg multiple testing correction in R version 3.0.2 using “stats” statistical package. Results with corrected *p*-values (*q*-values) < 0.05 were considered statistically significant.

## Author Contributions

A.R. participated in the intellectual design of the study, carried out the treatment of neuroblastoma cell lines with nutlin-3, performed the miRNA profiling, viability experiments, differentiation assays and western blot, participated in the ChIP-qPCR, and drafted the manuscript. A.V.G. participated in the miRNA profiling. M.O.N. analysed publicly available p53 ChIP-Seq data. S.D.B. participated in the isolation of the neuroblasts. P.J.V. designed the primers of the ChIP-qPCR. S.A. and J.S. performed the p53 ChIP. K.D.P. participated in the isolation of the neuroblasts, and analysed the survival data and the expression data of the miRNAs. P.M. participated in the miRNA profiling and analysis of the miRNA expression data. F.S.P. participated in the intellectual design of the study. J.V. participated in the intellectual design of the study. T.V.M. participated in the intellectual design of the study, supervising, and assisted in drafting, critical revising of the manuscript and gave the final approval for submission. All authors reviewed the manuscript.

## Supplementary Material

Supplementary Information supplementary information
