# Supplementary material for: Genome wide expression profiling of p53 regulated miRNAs in neuroblastoma
Source: Sci Rep. 2015 Mar 12;5:9027. doi: 10.1038/srep09027 (PMC4356961; doi:10.1038/srep09027)
Supplement: Supplementary Information [file srep09027-s1.pdf]

Genome wide expression profiling of p53 regulated miRNAs in neuroblastoma

Ali Rihani[1], Alan Van Goethem[1], Maté Ongenaert[1], Sara De Brouwer[1], Pieter-Jan Volders[1], Saurabh Agarwal[2], Katleen De Preter[1], Pieter Mestdagh[1], Jason Shohet[2], Frank Speleman[1], Jo Vandesompele[1], and Tom Van Maerken[1,§]

[1]Center for Medical Genetics, Ghent University, De Pintelaan 185, B-9000 Ghent, Belgium

[2]Texas Children's Cancer Center, Baylor College of Medicine, Houston, Texas

[§]Corresponding author

Figure S1

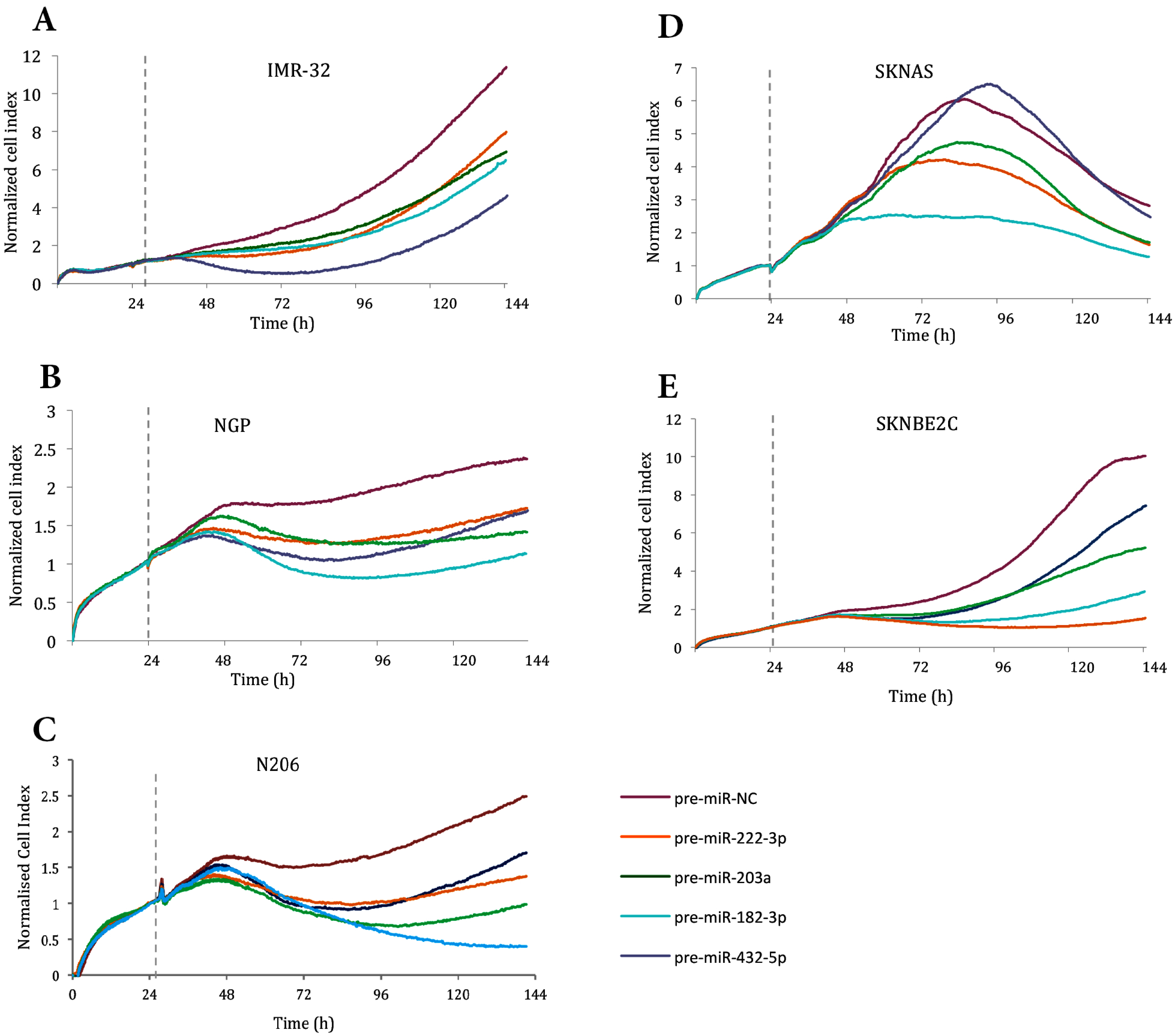

A
IMR-32
Normalized cell index
0
2
4
6
8
10
12
24
48
72
96
120
144
Time (h)
B
NGP
Normalized cell index
0
0.5
1
1.5
2
2.5
3
24
48
72
96
120
144
Time (h)
C
N206
Normalised Cell Index
0
0.5
1
1.5
2
2.5
3
0
24
48
72
96
120
144
Time (h)
D
SKNAS
Normalized cell index
0
1
2
3
4
5
6
7
24
48
72
96
120
144
Time (h)
E
SKNBE2C
Normalized cell index
0
2
4
6
8
10
12
24
48
72
96
120
144
Time (h)
pre-miR-NC
pre-miR-222-3p
pre-miR-203a
pre-miR-182-3p
pre-miR-432-5p

Figure S2

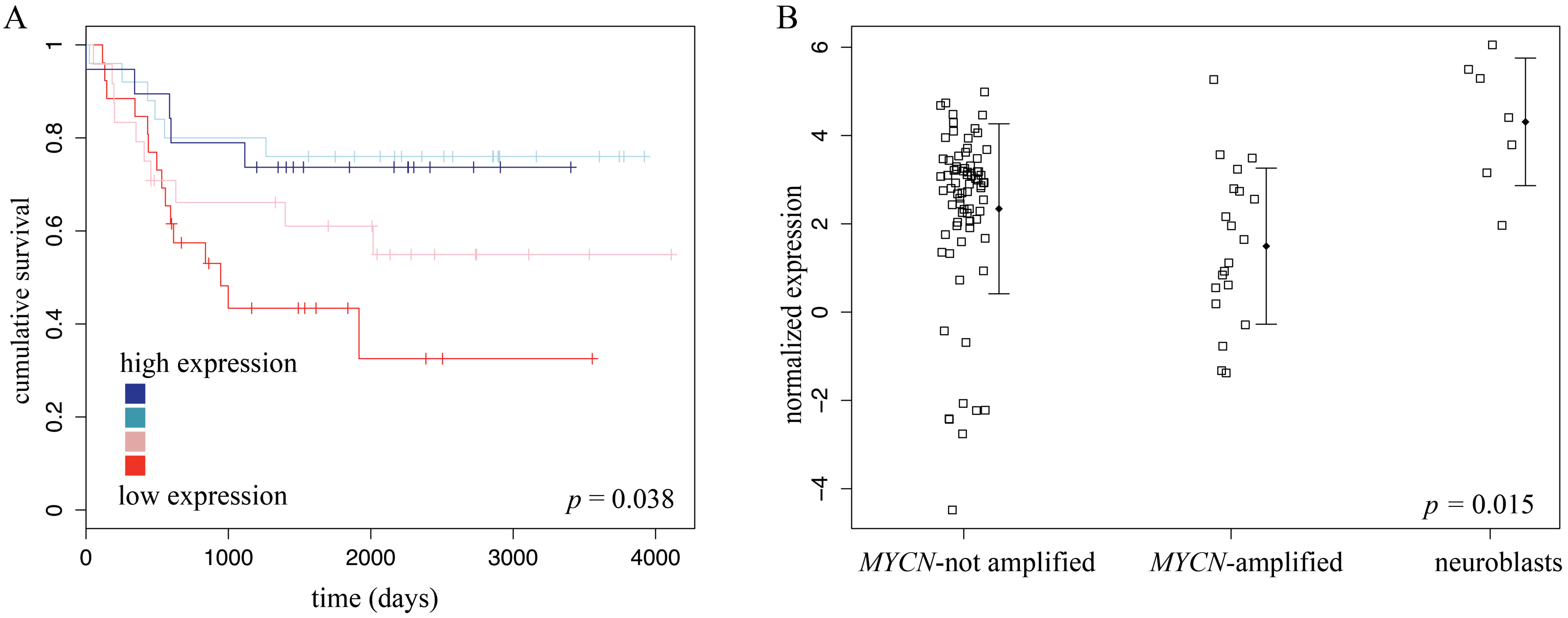
A
cumulative survival
1
0.8
0.6
0.4
0.2
0
high expression
low expression
p = 0.038
0
1000
2000
3000
4000
time (days)
B
normalized expression
6
4
2
0
−2
−4
p = 0.015
MYCN-not amplified
MYCN-amplified
neuroblasts

Figure S1: **Overexpression of miRNAs reduces proliferation.** Neuroblastoma cell lines were transfected with the indicated pre-miRs, and their growth was assessed using the xCELLigence system. Transfection time point is used for normalization (dotted line). IMR-32(**A**), NGP (**B**), N206 (**C**), SKNAS (**D**), SKNBE(2c) (**E**).

Figure S2: **Survival and expression analysis of miR-432-5p.** Kaplan-Meier progression-free survival curve for neuroblastoma patients (n=100) stratified according to miR-432-5p expression levels (quartiles) (log-rank test p-value) **(A)**. Comparison of miR-432-5p expression between neuroblasts and primary neuroblastoma tumors stratified according to MYCN status, data compared using rank product analysis. MNN (*MYCN* non-amplified neuroblastoma), MNA (*MYCN* amplified neuroblastoma) **(B)**.

miR-432-5p expression is associated with progression free survival of neuroblastoma patients and is expressed higher in normal fetal neuroblasts

We analyzed the expression patterns of the aforementioned miRNAs in a cohort of 100 primary neuroblastoma tumors and human fetal adrenal neuroblasts (details in materials and methods), the latter being the cell of origin for neuroblastoma [7,12]. While the miRNAs were not significantly associated to overall survival, low expression of miR-432-5p was significantly associated with poor progression-free survival and shows lower expression in the *MYCN*-amplified neuroblastoma tumors as compared to the tumors without *MYCN* amplification. When comparing the expression of the 4 miRNAs between the neuroblastoma tumors and the neuroblasts, miR-432-5p and miR-203a show higher expression in the neuroblasts as compared to the neuroblastoma tumors (Figure 4).
